# Channel-Based Key Generation for Encrypted Body-Worn Wireless Sensor Networks

**DOI:** 10.3390/s16091453

**Published:** 2016-09-08

**Authors:** Patrick Van Torre

**Affiliations:** Department of Information Technology, Ghent University/iMinds, Technologiepark-Zwijnaarde 15, 9052 Gent, Belgium; patrick.vantorre@ugent.be; Tel.: +32-9-331-4882

**Keywords:** body-centric communication, wireless sensor networks, key generation, encryption

## Abstract

Body-worn sensor networks are important for rescue-workers, medical and many other applications. Sensitive data are often transmitted over such a network, motivating the need for encryption. Body-worn sensor networks are deployed in conditions where the wireless communication channel varies dramatically due to fading and shadowing, which is considered a disadvantage for communication. Interestingly, these channel variations can be employed to extract a common encryption key at both sides of the link. Legitimate users share a unique physical channel and the variations thereof provide data series on both sides of the link, with highly correlated values. An eavesdropper, however, does not share this physical channel and cannot extract the same information when intercepting the signals. This paper documents a practical wearable communication system implementing channel-based key generation, including an implementation and a measurement campaign comprising indoor as well as outdoor measurements. The results provide insight into the performance of channel-based key generation in realistic practical conditions. Employing a process known as key reconciliation, error free keys are generated in all tested scenarios. The key-generation system is computationally simple and therefore compatible with the low-power micro controllers and low-data rate transmissions commonly used in wireless sensor networks.

## 1. Introduction

Wearable wireless sensor networks are a convenient solution to communicate measurement results from person to person, or from a person to a base station. The data often include sensitive information such as coordinates or medical data, requiring secure transmission. With the current evolution of the Internet of Things (IoT) and the Internet of People (IoP), secure wearable wireless sensor networks are gaining even more importance [1].

Given the limitations imposed by the hardware of typical wireless sensor nodes, there is a need for encryption protocols that are energy efficient and require limited memory and processing power. Most conventional encryption schemes do not meet these demands, and also require considerable packet overhead in the form of header extensions. Therefore, current research focuses on lightweight block ciphers, which are, however, vulnerable to security issues as well as denial-of-service attacks [2]. Research into a potentially more secure scheme was recently presented in [3].

The wireless sensor nodes in this paper employ communication according to the IEEE802.15.4 standard, of which the MAC (Media Access Control) layer includes AES128 (Advanced Encryption Standard) with seven security levels, of which the highest level combines 128 bits AES encryption and 128 bits MIC (Message Integrity Code). However, key management and generation algorithms are not included and should be provided by the upper network layer. It is exactly the key distribution that is challenging and current techniques are often based on the predistribution of secret material, causing large memory overhead. A more memory efficient scheme is proposed and analyzed in [4], but not practically implemented. Encryption employing AES-CCM-128 (Standard-Counter with Cipher Block Chaining-Message Authentication Code) requires less packet overhead but needs dedicated hardware support to be energy-efficient [5]. However, another very interesting approach for symmetric key generation is possible, without exchanging information over the wireless channel, if a common private source of information can be used for key generation. Such an approach has the potential to make symmetric key generation more secure compared to the protocols mentioned above.

The idea of having a common and unique source of information for key generation has also been studied for body area sensor networks before [6], where biometric signals captured by different nodes on the same person are used as a source of common information.

The source of common information for body-worn wireless sensor nodes originates from the properties of the radio propagation channel. Wearable sensor networks are typically deployed in environments where the communication channel varies significantly due to fading and shadowing [7]. In the case of off-body communication, changing antenna orientation and additional shadowing by the human body also play an important role. Recent work documented in literature suggests that the varying channel can be employed to generate secure keys via a novel approach [8,9].

Legitimately communicating users share a unique physical propagation channel of which the characteristics can be measured at both sides of the link. The channel measurements are highly correlated for the legitimate users and the mutual information between both sets of data allows to extract similar keys by both users. Some key errors might occur, but reconciliation techniques are further applied to successfully adjust the keys.

An eavesdropper, however, does not share the same physical channel, and eavesdropper measurements will typically be decorrelated from the legitimate users’ data. Mutual information between the data series will also be very limited [10], rendering key estimation by the eavesdropper cumbersome. Measurements using a network analyzer confirmed the usefulness of channel-based key-generation before, as documented in [8,9]. The influence of the radio-wave propagation channel onto key security has recently been further analyzed and documented in [11].

This paper discusses practical research on the performance of a wearable channel-based key-generating system, employing realistic body worn sensor nodes, of which the hardware and network performance were documented earlier [12]. The embedded software in the nodes has been modified to exchange packets between legitimate parties within a very narrow time slot, allowing the accurate estimation of the channel at both ends of the link. The raw Key Error Rate (KER) is determined and a threshold is employed to reduce the KER below a level for which further reconciliation yields error free keys.

The proposed and tested key generation algorithm is very suitable for body-centric sensor networks and allows secure key generation without additional hardware. Encryption by means of the generated key can be performed employing a number of algorithms suitable for execution on a low-power micro controller [13]. Keys generated following this approach can be constantly updated and hence should be virtually impossible to break, provided that careful hardware construction of the sensor node makes side-channel attacks (by measuring other electrical parameters such as power supply current) impossible [14]. Emissions by the processor should be minimized, as advanced side-channel attacks are possible with very low cost Software Defined Radios (SDR) [15]. In our design, the very small footprint low-power micro controller is shielded by the wearable antenna’s ground plane as well as by the human body.

Wearable sensor nodes benefit from the natural movement of the body to create sufficient channel variation for low-bitrate key generation, without the need for re-configurable antennas [16] or multi-antenna configurations [17]. However, the key-generation rate can be increased using multiple on-body antennas, as the human body provides a large platform to deploy multiple antennas with limited mutual coupling [18].

To the author’s knowledge, this is the first fully functional implementation of this type of key generation on wearable autonomous wireless sensor nodes. Initial work was documented in [10,19], but now, full key generation is performed for indoor as well as outdoor scenarios, and the results are documented, compared and analyzed more in depth.

## 2. Materials and Methods

### 2.1. Wireless Sensor Nodes

#### 2.1.1. Hardware

**Figure 1:**
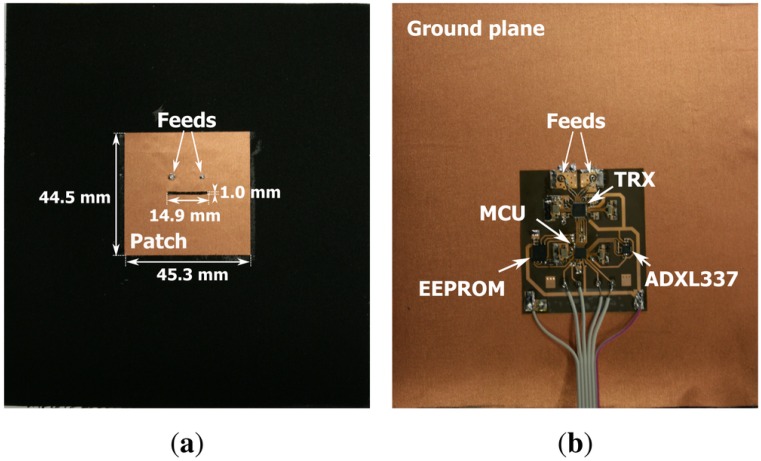
(**a**) front view of the wireless sensor node; (**b**) back side of the wireless sensor node with the electronic components mounted on the flexible substrate.

The wireless sensor nodes, employed to collect the channel measurements, are fully integrated flexible wearable wireless sensor nodes, as shown in Figure 1. A front view of the antenna is shown in Figure 1a, displaying the radiating patch with two feeds for dual-polarized wave excitation [20]. The backside of the antenna is shown in Figure 1b, depicting the circuitry integrated on the ground plane. All the hardware of a fully functional wireless sensor system is integrated onto the back of the highly flexible textile patch antenna. The hardware includes a C8051F920 low-power micro controller unit (MCU) (Silicon Labs, Austin, TX, USA), an ADF7242 transceiver (TRX) chip (Analog Devices, Norwood, MA, USA), an Electrically Erasable Programmable Read-Only Memory (EEPROM) (Microchip Technology, Chandler, AZ, USA) and an ADXL337 three-axis accelerometer (Analog Devices, Norwood, MA, USA), mounted on a flexible polyimide substrate. Other analog or digital sensors can be easily connected or integrated in a redesign of the circuit. The wireless sensor node is able to perform fully synchronous measurements on all nodes in the network. Measurement data is forwarded between nodes worn at different locations on the same body or worn by different persons operating within a range of up to 50 m in Non Line-of-Sight (NLoS) radio propagation conditions. Further details of the hardware are documented in [12].

#### 2.1.2. Reciprocal Channel Estimation

In order to perform measurements for channel-based key generation, the software on the wireless sensor nodes is adapted to bilaterally assess reciprocal channel parameters in a very short time slot. The ADF7242 TRX, in combination with the C8051F920 MCU, allows a transmit-receive turnaround time of 5 ms between legitimate users.

**Figure 2:**
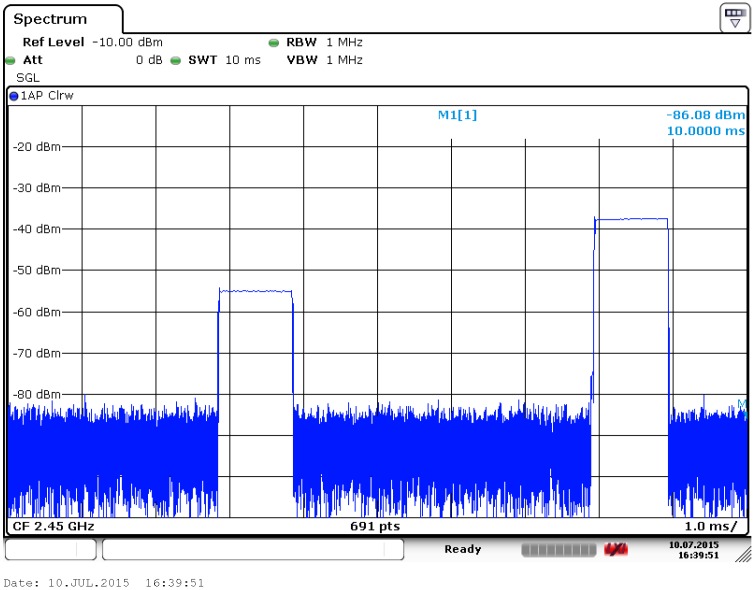
Rohde & Schwarz FSV40 Screenshot for a bilateral channel estimation transmission with 5 ms transmit-receive turnaround time.

A bilateral transmission by the system for channel measurements in the 2.45 GHz Industrial, Scientific and Medical (ISM) band, as picked up by a Rohde & Schwarz FSV40 Signal Analyzer (Munich, Germany), is displayed in Figure 2. All transmissions are performed in IEEE802.15.4 format, relying on Direct Sequence Spread Spectrum (DSSS) techniques, with a transfer rate of 250 kbit/s. The transmissions contain a Cyclic Redundancy Check sum (CRC). Packets are filtered by the receiver hardware based on a correct CRC, making the probability of errors so extremely low that all received packets should be considered error free.

The horizontal scale is 1 millisecond per division, whereas the vertical scale is the received signal power in dBm. Clearly, transmissions last 1 ms and are separated by only 5 ms. Note that this is a reciprocal channel estimation with the second transmission being the answer from the other side of the link, upon error free reception of the first transmission.

The system enables reciprocal channel estimation within the coherence time of the channel, implying that the channel variation within the 5 ms time frame is known to be limited. As a consequence, the bilaterally measured values for the Received Signal Strength (RSS), will be highly correlated and share significant mutual information. The resolution of the RSS measurement is 1 dB, but imbalance due to hardware tolerances can make channel estimation less accurate.

An additional source of tolerances on the channel measurements occurs due to the nature of the radio propagation. The main cause of the signal variation is small-scale fading, caused by the changing conditions of constructive or destructive interference between multiple reflected radio waves. Destructive interference causes an RSS minimum, appearing as a notch when presented graphically. At a moment of destructive interference, the channel varies very fast, increasing the difference between reciprocal measurements within the 5 ms time slot.

### 2.2. Measurement Setup

Throughout the paper, the names Alice, Bob and Eve will be used, according to existing literature in the field of channel-based key generation [9]. Alice and Bob are the legitimately communicating parties, simultaneously moving around, whereas Eve is the stationary eavesdropper, trying to assess the keys used by Alice and Bob.

#### 2.2.1. Measurement Environments

**Figure 3:**
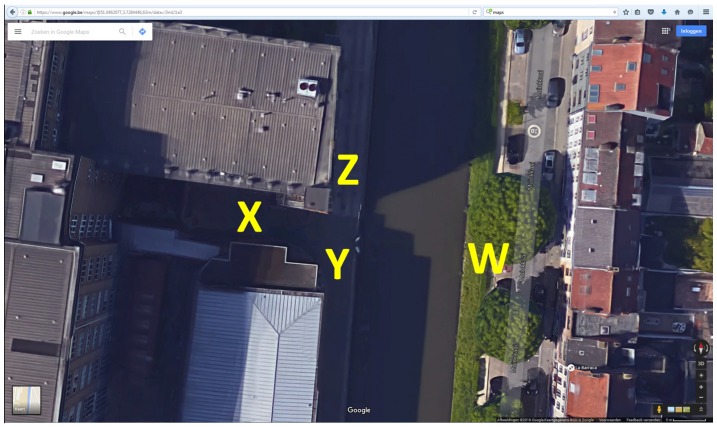
Outdoor measurement area, from Google Maps.

Indoor as well as outdoor measurements have been performed. Outdoor propagation measurements were also performed over larger distances, across the canal visible in Figure 3. The positions at which the communicating parties are located are indicated by letters. Alice and Bob perform random walks, taking care to stay within the intended propagation scenario, as listed further. Eve is always in a fixed position.

Propagation can occur LoS (Line-of-Sight) when no buildings or obstacles are blocking the radio waves between communicating parties. In NLoS (Non Line-of-Sight) outdoor propagation, the line-of-sight path is obstructed by buildings and the wireless link is established mainly via reflections and scattering in the environment.

**Figure 4:**
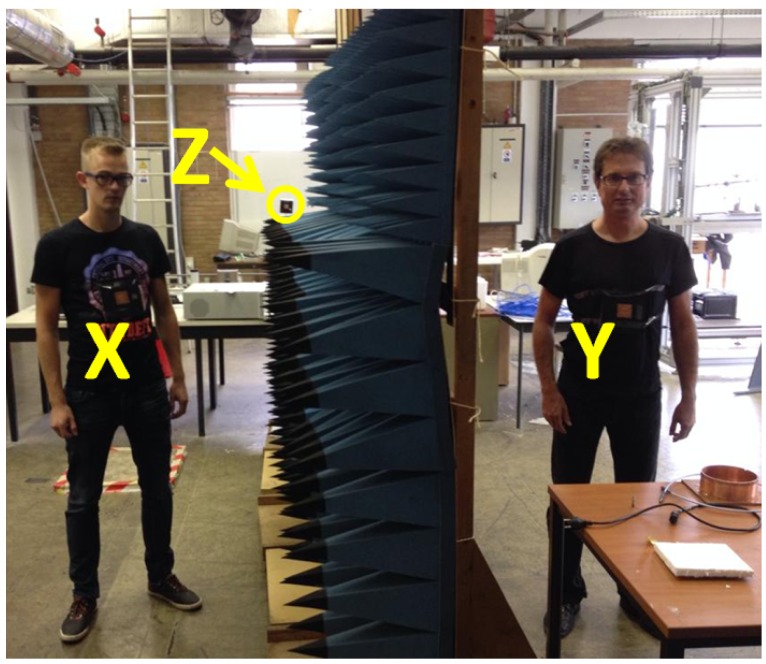
Indoor measurement area, Alice-Bob Non Line of Sight, Eve Line of Sight to both legitimate parties, Scenario 7. Letters indicate indoor positions.

Indoor radio propagation environments characteristically have much more multipath propagation, leading to different fading characteristics and more complex channel variation. As visible in Figure 4, lots of infrastructure can lead to reflections and hence complex multipath propagation. Reflections occur at walls, the ceiling and floor, furniture and of course metal structures. Here, Alice and Bob are in positions X and Y, respectively, whereas Eve is in position Z, corresponding to Scenario 7, as further outlined.

The picture also clearly displays the compactness and wearability of the system, where the wireless sensor nodes are easily integrated onto a T-shirt without any external wired connections, retaining a high flexibility. Eve is a similar node mounted in a fixed position. In the picture, Eve’s position is indicated by the arrow at location Z.

#### 2.2.2. Measurement Scenarios

The measurement scenarios are listed in Table 1. Scenarios are numbered, for further discussion. Indoor or outdoor propagation is marked as well as the type of propagation in terms of LoS or NLoS conditions between all communicating parties. The positions at which the communicating parties operate to obtain a specific scenario are indicated in the last three columns of the table. The letters refer to positions in Figure 3 for the outdoor scenarios, and in Figure 4 for the indoor scenarios.

For both indoor and outdoor environments, two regular scenarios are tested, being Alice and Bob in LoS, with Eve either LoS or NLoS. These are the most realistic scenarios to occur, with an eavesdropper inside or outside the building for the indoor case, or hidden behind a building or not for the outdoor case. For the outdoor case, a measurement is also performed with the legitimate parties across the canal in Figure 3, with respect to the eavesdropper.

A worst-case scenario is also tested for both the indoor as well as the outdoor environment, in which the legitimate parties Alice and Bob are NLoS, but with direct LoS from both parties to the eavesdropper, Eve. This situation is unlikely to occur in practice but is interesting to analyze because, with NLoS propagation between Alice and Bob, reflections in the vicinity of Eve will likely contribute to the propagation between the legitimate parties. Therefore, channel measurements performed by Eve in this scenario are expected to be slightly more correlated to the measurements of Alice and Bob.

#### 2.2.3. Processing of the Channel Measurements

The transceiver chip performs channel measurements by means of an analog logarithmic detector, which are further sampled as 8-bit values, directly expressed in dB. The resolution of the measurement is 1 dB, according to the datasheet. Inaccuracy can result from channel variation, chip-to-chip parameter spread, or system imbalances such as differing antenna performance and matching. Hence, the bytes that result from the reciprocal detectors are generally not equal, despite their high correlation.

To design a system that functions in practice by exploiting these channel measurements, a quantizer is applied at Alice’s side to extract only one bit per channel measurement according to the following procedure:

- Determine the moving average of the last seven RSS values (corresponding to the average over the last 7 s) [10].
- If the current RSS value crosses a threshold of *N* dB above or below this value, a 1 or a 0 bit is generated, respectively. Bob is informed about the generation of a key bit via the wireless channel, without revealing the actual value of the bit.
- If the threshold is not crossed, no bit is generated.

The quantizer at Bob’s side also extracts one bit per channel measurement, as follows:

- Determine the moving average of the last seven RSS values.
- If Bob is informed by Alice that she has generated a key bit, Bob also generates a key bit:
  - A 1-bit is generated if Bob’s measured RSS value is above the threshold
  - A 0-bit is generated otherwise

If Alice generates the master key, she will choose and apply the threshold level. Each time Alice uses an RSS value to generate a key bit, she will immediately inform Bob over the wireless link. The information of course does not contain the actual value of Alice’s key bit. Bob can now generate his own version of the key bit, based on his own RSS measurement. This approach is absolutely necessary in order for Alice and Bob to use sets of RSS values that are sampled in corresponding time slots.

Independently choosing RSS samples on both ends of the link via equal thresholds does not work in practice. Due to the imperfect reciprocity of the measurements, the generated keys would quickly run out of synchronization since, at some time instances, only one party would generate a key bit.

Note that the analysis presented in this paper is based on the worst-case assumption that Eve knows all the details about the system and is able to intercept all the information that Alice and Bob exchange, including knowledge about the time slots where Alice and Bob select RSS samples. All calculations have been performed to be consistent with this assumption.

Only one RSS measurement per second is presented to the quantizer, in order to obtain subsequent random bits that are sufficiently decorrelated. The time interval of one second corresponds well to the rate of change of the RSS values recorded for normal walking speeds of about 0.5 m/s. Faster walking speeds could allow faster key generation, but, at the moment, the rate of which the RSS values are presented to the quantizer is kept fixed. Employing higher thresholds further limits the key generation rate because more measurements are dropped. However, higher thresholds will result in better matching between bilateral raw keys.

The bilateral set of raw keys needs further reconciliation to exactly match. The (11,7)-Hamming forward error correcting code is employed to achieve equal keys. After pseudo-random bit interleaving, Alice’s raw key is taken as the master key and subdivided into 11-bit groups. For each group, four Hamming check bits are calculated and transmitted to Bob. The potential key errors in Bob’s key are corrected based on the check bits from Alice’s key. As a last step, de-interleaving is performed to undo the interleaving. Interleaving scrambles bits in pseudo-random order to spread groups of subsequent key errors over a large number of Hamming words, improving the performance of the error correcting code.

Transmitting the check bits reveals some information about the key to a potential eavesdropper, assuming the unlikely fact that the eavesdropper has all detailed information about the complete key-generating system. Employing the Hamming code and revealing four check bits, results in $2^{7}$ valid 11-bit words. The effective key length is hence reduced by a factor 7/11 if the eavesdropper performs a brute-force attack with knowledge of the check bits. However, even then, a sufficiently long and regularly updated key cannot be reconstructed by an eavesdropper.

## 3. Results

### 3.1. Correlation for Legitimate Parties versus Eavesdropper

**Figure 5:**
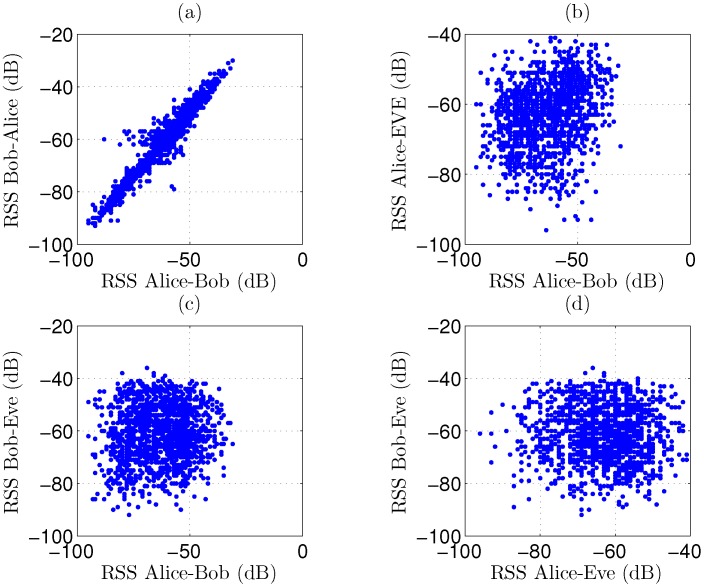
Scatterplots for 1500 recorded RSS (Received Signal Strength) channel samples in scenario 1, displaying high correlation for the legitimate parties and very low correlation for the eavesdropper. (**a**) Correlation for Bob-to-Alice versus Alice-to-Bob signals; (**b**) Correlation for Alice-to-Eve versus Alice-to-Bob signals; (**c**) Correlation for Bob-to-Eve versus Alice-to-Bob signals; (**d**) Correlation for Bob-to-Eve versus Alice-to-Eve signals.

**Figure 6:**
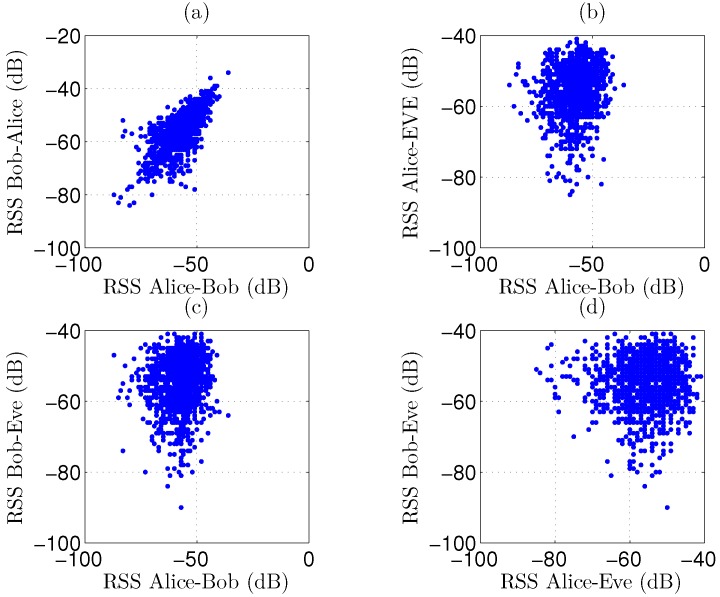
Scatterplots for 1500 recorded RSS channel samples in scenario 7, displaying slightly reduced correlation for the legitimate parties and very low correlation for the eavesdropper. (**a**) Correlation for Bob-to-Alice versus Alice-to-Bob signals; (**b**) Correlation for Alice-to-Eve versus Alice-to-Bob signals; (**c**) Correlation for Bob-to-Eve versus Alice-to-Bob signals; (**d**) Correlation for Bob-to-Eve versus Alice-to-Eve signals.

As a first observation, 1500 RSS channel samples for Scenario 1 are displayed in Figure 5, for the reciprocal link between the legitimate parties as well as for the link from Alice to Bob. These results are compared to the signal levels captured by Eve. A very strong correlation is observed in Figure 5a for reciprocal channel measurements between the legitimate parties, as the measurement pairs are clustered, resembling a rising straight line with a positive slope. In plots Figure 5b,c, a very low correlation is present between the legitimate link and the signal levels intercepted by the eavesdropper, resulting in a more circular cloud. Scatter plot Figure 5d shows the correlation between Alice’s and Bob’s signals, as captured by the eavesdropper. From these graphs, we can conclude that distilling useful data from channel measurements is difficult for the eavesdropper. Results are very similar for scenarios 2 to 6. In the worst-case scenario 7, correlation for the legitimate parties is less strong due to NLoS propagation between them, as clearly visible in Figure 6. Although the correlation is still very low for the eavesdropper, scenario 7 appears more difficult for key generation.

### 3.2. Legitimate Key Generation

**Figure 7:**
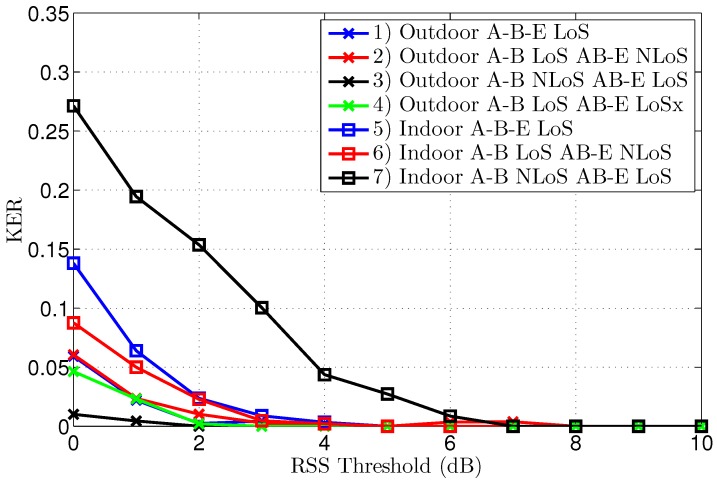
Key Error Rate after reconciliation for different threshold levels, for all scenarios.

**Figure 8:**
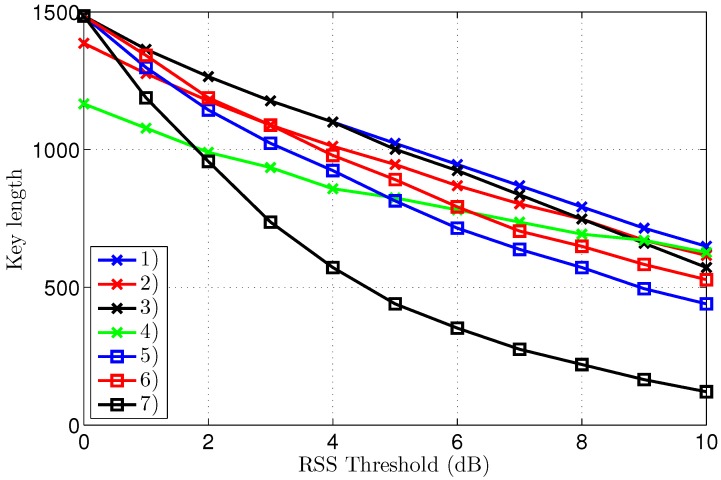
Key Error Rate after reconciliation for different threshold levels, for all scenarios.

The KERs (Key Error Rates) and the corresponding key lengths after reconciliation are displayed in Figure 7 and Figure 8, respectively. To clearly explain the results, both graphs will be discussed at the same time. A further analysis of the interpretation of these results follows in the discussion section.

The first important observation is that the KER converges to zero for all scenarios, provided a significantly large threshold is employed, clearly illustrating effective key generation in all tested conditions. Without threshold, or with thresholds of only 1 or 2 dB, the KER is systematically lower for the outdoor scenarios. The point of zero bit errors is achieved at a threshold of only 2 dB for scenario 3, resulting in an error-free key length of 1364 bits, based on 1500 channel samples. For all other scenarios, except scenario 7, a threshold of 5 dB is necessary to obtain a KER of zero, making the performance for indoor and outdoor scenarios comparable. For the worst-case scenario 7, manifesting also the highest initial KER, a zero KER is obtained only at a threshold level of 7 dB.

Note that a further increase of the threshold beyond the first zero KER point can coindicentally result in some key errors again, which is the case for scenario 2, but for a higher threshold, the KER finally converges to zero.

The key length in Figure 8 decreases for higher thresholds, as larger parts of the channel measurements are eliminated from the data set. For equal thresholds and similar propagation conditions in terms of LoS or NLoS, the key generation rate is systematically higher for the outdoor scenarios. The smallest key length of 275 bits results for scenario 7 at a threshold of 7 dB. At the same threshold level, the key length is still 869 bits for scenario 1. As 1500 channel measurements were collected for both scenarios, at a rate of one measurement per second, the key generating rate is 0.6 bits/s for scenario 1, compared to 0.2 bits/s for scenario 7 at the same 7 dB threshold. However, for scenarios 1–6, the first point of zero KER already occurs at a threshold level of 5 dB, corresponding to a key length up to 1023 bits or up to a key generation rate of 0.7 bits/s.

To obtain the highest possible key generating rate, the lowest possible threshold should be used. Detecting the point of error-free keys can be easily performed by exchanging a CRC (Cyclic Redundancy Check) sum on the key bits between the legitimate parties.

Note in the graph that, for scenarios 2 and 4, the key length without threshold is a bit shorter than 1500 bits, due to packet loss over the difficult link to Eve, which is either NLoS or larger distance LoS. In the analysis, only packets received successfully by all three nodes are considered.

### 3.3. Attempted Eavesdropper Key Generation

It is equally important to study the behavior for key generation attempted by Eve, assuming the worst-case situation that Eve captures signals from both Alice and Bob, knows all details about the system and is also able to intercept the check bits.

Eve tries to estimate the master key based on recorded signal levels. Four cases are possible for the measurement discussed here:

1. Alice has the master key, Eve uses Alice’s signal strength
2. Alice has the master key, Eve uses Bob’s signal strength
3. Bob has the master key, Eve uses Alice’s signal strength
4. Bob has the master key, Eve uses Bob’s signal strength

All cases have been studied in detail, but we document only graphs for the worst of these four cases, where key extraction by Eve reveals the most information about the key. This corresponds to case number 1 in the list.

**Figure 9:**
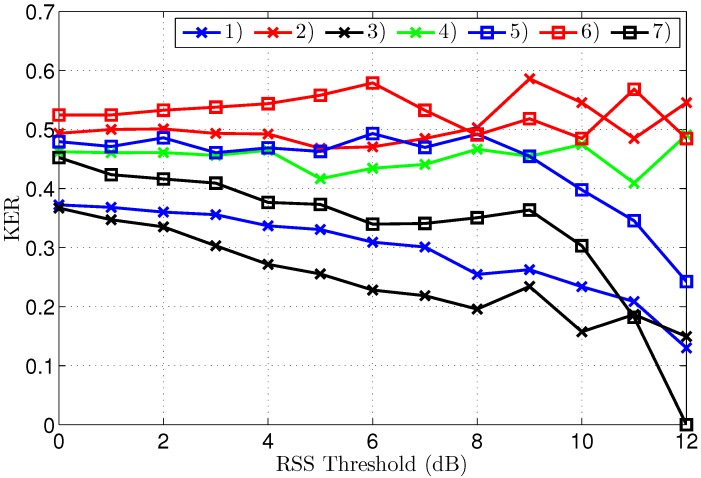
Key Error Rate after reconciliation for different threshold levels, for all scenarios.

Figure 9 displays the KER after reconciliation by Eve for different thresholds. Clearly the KER does not converge to zero as easily as for the legitimate parties. The KER for scenarios 2, 4 and 6 does not approach zero at all. The KER is finally decreased for higher thresholds in the other scenarios, but stays too high to be of practical use, except for the worst-case scenario 7.

It is important to note that convergence to a zero KER at a threshold of 12 dB for scenario 7 does not mean that Eve can intercept the key used by Alice and Bob. The legitimate parties normally choose to operate at a threshold level of 7 dB for this scenario, as this is the lowest threshold resulting in a zero KER for them. At this threshold level, the KER for Eve is still 0.34, which is very high, considering that a KER of 0.5 corresponds to no information at all.

**Figure 10:**
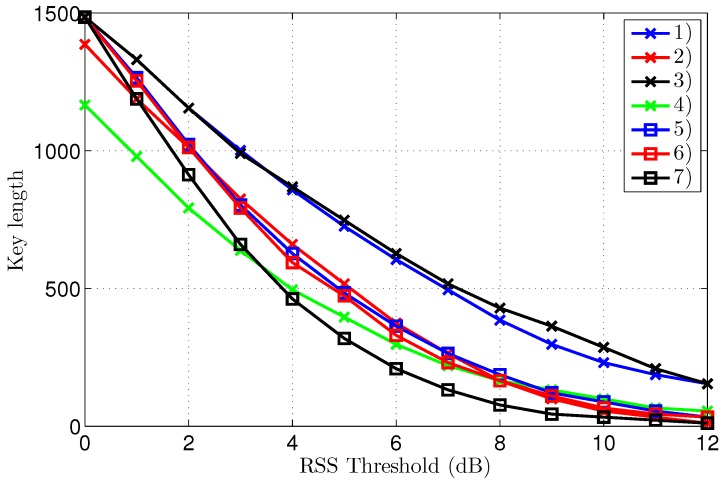
Key Error Rate after reconciliation for different threshold levels, for all scenarios.

Further reasons why convergence to lower KER values for Eve is not of practical use follows from the key length shown in Figure 10. For higher threshold levels, the key length quickly becomes impractically short, implying that, even in the single scenario where Eve can construct an error-free key, that key is much shorter than the key used by Alice and Bob, which is constructed at the lower threshold level of 7 dB instead of 12 dB. Actually for our measurement in scenario 7, Eve’s error free key is only 11 bits long, corresponding to a single Hamming code word. Note that, in the same scenario, Alice and Bob use a 275-bit key. Constructing keys at a different threshold level than the one used by Alice and Bob is actually pointless, as these keys do not match in both length and content.

## 4. Discussion

### 4.1. Key Generation Performance

Error-free key generation is possible in all tested scenarios. However, due to differences in the statistics of the signal levels, the performance differs for the different scenarios. To obtain error-free keys after reconciliation, a threshold of 5 dB is sufficient for six out of seven scenarios. A comparison of the key generating rate at this threshold level is therefore most relevant. Note that the threshold value yielding error-free keys is determined automatically by exchanging a CRC calculated for the key which is generated after reconciliation. A valid CRC indicates matching keys at both sides of the link, allowing both parties to select an equal threshold level, which is as low as possible to obtain error-free keys.

Observed key lengths extracted from the measurements are clearly the largest for the outdoor scenarios, despite the presence of some packet loss in two outdoor scenarios. Outdoor keys have a smaller variance, with a length between 836 and 1001 bits. For the indoor scenarios, the key length varies between 429 and 792 bits. The reason for this performance difference can be found in the physical channel behavior, which is different for indoor and outdoor areas. Three main sources of differing reciprocal channel measurements are identified:

- Limited resolution of the RSS detector
- Hardware imbalances of the TRX and the antennas
- Channel small-scale fading characteristics

The first two sources of errors are always present, regardless of the scenario. However, the third source is much more dominant for indoor scenarios, for which more complex propagation conditions exist [7]. In indoor environments, much more multipath components influence the received signal strength, resulting in faster channel variation for similar walking speeds. Sporadically occurring extremely fast channel variations during the fading minima create a random difference in reciprocal channel measurements within the 5 ms time slot, directly causing raw key errors.

For scenario 7, a higher threshold of 7 dB is necessary, decreasing the key length to 275 bits. It is important to realize that the loss in key-generation rate, caused by applying the necessary threshold, can be significantly reduced if the transmit-receive turnaround time of the system is decreased to 1 ms or lower. Hardware with less tolerance and a better resolution could finally render the raw KER, before thresholding and reconciliation, very low. In the measurement campaign, the performance is limited due to the state-of-the-art of currently available off-the-shelf components.

### 4.2. Performance of Eavesdropper Key Generation

Key generation by the eavesdropper is shown to be cumbersome. Eve cannot extract useful keys from her channel measurements. However, some differences in the results exist, dependent on the propagation scenario. For scenarios 2, 4 and 6, the KER for the eavesdropper does not converge at all. A decrease in KER is observed at higher threshold levels for the other scenarios. Increasing the threshold quickly makes the key shorter, therefore a low, or even zero KER obtained by Eve is not useful, as the number of effective key bits is very low. Moreover, the legitimate parties operate at lower threshold levels, for which the KER for Eve is always above 0.2 and often much higher. Even if Eve were able to use the few bits collected at a higher threshold level to estimate some bits of the longer keys employed by Alice and Bob, those bits only represent a very small part of the legitimate key, corresponding to 4% of the legitimate key bits in the worst-case scenario.

### 4.3. Choosing a Practical Threshold Level

In a practical system, the threshold should be chosen at the moment of key generation. The measurements provide an indication about which threshold values are realistic. A threshold of 8 dB provides a zero KER in all scenarios of the measurement campaign. Given the number of scenarios tested, a fixed threshold of 10 dB is likely to produce error free keys in nearly all cases. Of course, this fixed threshold does not lead to the highest possible key-generation rate.

For Alice, a more sophisticated option is to dynamically adapt the threshold, based on feedback about the correctness of the key. Bob can transmit this feedback to Alice as a CRC check sum over a number of key bits generated by him after reconciliation. Alice can hence dynamically adjust her threshold level and set it as low as possible for error-free key generation by Bob. This approach should allow a larger average key generation rate for all scenarios.

### 4.4. Guaranteeing a Secure System Start up

To make the communication secure from the start, a predefined key should be used initially. In practice, a random initial key can be securely distributed to all the nodes, via a wired connection to a common battery-charging unit. It is highly recommended not to send unencrypted packets at the startup process, as this will allow a potential eavesdropper to analyze the system. Note that the results in this paper are actually for an eavesdropper who does know all these details of the system and intercepts all packets. However, encrypting packets from the start makes it virtually impossible for an eavesdropper to extract these details, further improving the security of the communication.

### 4.5. Increasing the Key Generation Rate

the largest restriction to increase the key generation rate is the limited channel measurement rate imposed by the time correlation of subsequent channel measurements. For walking persons, channel measurement rates much higher than one sample per second will yield low-entropy keys.

The channel measurement rate can further be improved by employing multi-antenna systems. Systems of *N* body-worn antennas create an N×N MIMO (Multiple-Input Multiple-Output) communication channel between two walking persons. In an N×N MIMO link, $N^{2}$ physical propagation paths exist, of which the varying parameters can be measured, potentially increasing the key-generation rate by a factor $N^{2}$. Note that such a system can obtain this performance by simply switching between antennas, hence full MIMO transmit-receive chains are not necessary, allowing faster key generation at a limited cost.

## 5. Conclusions

A measurement campaign for channel-based key generation was performed, with indoor as well as outdoor measurements. A set of seven scenarios was tested, including worst-case setups in difficult conditions. Successful key generation was always possible for the legitimate parties, with key generating rates slightly higher for the considered outdoor scenarios. Interception of the keys by the eavesdropper is impossible as illustrated from the measurements. In case the eavesdropper knows all the details of the system and intercepts all packets, only very limited information about the key could be retrieved in the worst-case scenario, potentially slightly decreasing the time needed for a successful brute force attack.

## Figures and Tables

**Table 1:** Outdoor and indoor Line-of-Sight (LoS) as well as Non Line-of-Sight (NLoS) measurement scenarios.

Scenario		Indoor/Outdoor	Alice-Bob	Alice-Eve	Bob-Eve	Location		
Number	Type	Environment	Link	Link	Link	Alice	Bob	Eve
1	Regular	Outdoor	LoS	LoS	LoS	Y	Y	X
2	Regular	Outdoor	LoS	NLoS	NLoS	Z	Z	Y
3	Worst case	Outdoor	NLoS	LoS	LoS	X	Z	Y
4	Across canal	Outdoor	LoS	LoS	LoS	W	W	Y
5	Regular	Indoor	LoS	LoS	LoS	Y	Y	Z
6	Regular	Indoor	LoS	NLoS	NLoS	Y	Y	X
7	Worst case	Indoor	NLoS	LoS	LoS	X	Y	Z

## Abbreviations

AES: Advanced Encryption Standard
CCM: Cipher block Chaining Message
CRC: Cyclic Redundancy Check
DSSS: Direct Sequence Spread Spectrum
EEPROM: Electrically Erasable and Programmable Read-Only Memory
IoP: Internet of People
IoT: Internet of Things
ISM: Industrial, Scientific and Medical (band)
KER: Key Error Rate
LoS: Line of Sight
MAC: Media Access Control
MCU: Micro Controller Unit
MIC: Message Integrity Code
MIMO: Multiple Input Multiple Output
NLoS: Non Line of Sight
RSS: Received Signal Strength
SDR: Software Defined Radio
TRX: Transceiver
